# Enhancing multiple benefits of brownfield cleanups by applying ecosystem services concepts

**DOI:** 10.3389/fevo.2024.1286150

**Published:** 2024-02-23

**Authors:** Brooke Mastervich, Kelly Garbach, Matthew C. Harwell

**Affiliations:** 1 Gulf Ecosystem Measurement and Modeling Division, Gulf Breeze, FL, United States; 2 U.S. Department of Agriculture, National Institute of Food and Agriculture, Petaluma, CA, United States; 3 Pacific Ecological Systems Division, Newport, OR, United States

**Keywords:** brownfields, nature-based solutions, ecosystem services, environmental policy, environmental benefits

## Abstract

Brownfields are increasingly called upon to be transformed from potentially contaminated, often vacant properties into community assets that provide multiple benefits. Further, brownfields revitalization can provide critical opportunities and, particularly, nature-based solutions can enhance multiple ecological, human health, and economic benefits. Through a series of non-exhaustive surveys of existing examples of environmental benefits of cleanups, case study examples of brownfield cleanups achieving environmental benefits, and potential ecosystem services tools relevant to steps of a brownfields cleanup effort, we explore practical ideas for enhancing environmental benefits of brownfields cleanups by applying ecosystem services concepts. Examples of nature-based solutions, where appropriate, include the use of rain gardens, permeable pavements, green spaces, and the use of green technologies. Further, this article provides an overview of recent policy initiatives focused on nature-based solutions and enhancing ecosystem services in brownfields cleanup, revitalization, and reuse. Our goals are to increase the knowledge base on these opportunities and discuss how these concepts can be achieved through sharing success stories, making outreach materials accessible, and holding workshops to help successfully operationalize these concepts in a community’s visioning for upcoming revitalization projects.

## 1 Introduction

Finding the balance in decision-making between rapidly growing societal needs and the goal of preserving, restoring, or enhancing the benefits that nature provides to people to help with resilience and sustainability is an ever-growing challenge. Ecosystem services are “the conditions and processes through which natural ecosystems, and the species that make them up, sustain and fulfill human life” (Daily, 1997). Ecosystem services as a concept provides an opportunity to identify, quantify, and evaluate ecosystem condition, status, and change associated with a decision, and describe the provisioning of environmental benefits to people to inform alternative selection, trade-off analyses, and cost-benefit analyses to support environmental decision making (Fisher et al., 2009). In recent years, ecosystem services have been at the forefront of efforts focusing on providing environmental benefits for human needs. For example, cleaning up contaminated lands, whether for commercial, residential, recreational, or greenspace use can result in jobs created for local residents, increases in property value, and provision of other services that contribute to improving a community (O’Sullivan et al., 2017). One type of land cleanups are brownfield sites, which are typically abandoned, idle industrial properties that have potential for redevelopment once environmental contaminants are removed (BenDor et al., 2011). The benefits of revitalizing brownfield areas may include economic, social, and environmental benefits (Söderqvist et al., 2015). Strom (2018) provides an overview of brownfield cleanups in urban environments, focusing on the importance of bringing together a suite of stakeholders interested in urban development and environmental concerns.

The aim of brownfield revitalization is to ultimately support sustainable and efficient redevelopment and employment opportunities, and the process of remediating contaminated sites creates opportunities for pursuing environmental restoration and community revitalization of the land (Williams and Hoffman, 2020). Each component allows important opportunities to develop and apply approaches to enhance social, economic, and environmental benefits. Regardless of where a given brownfield site is in the overall cleanup process (pre-development, development, management) or ultimate endpoint (remediation, restoration, or revitalization), understanding both economic and environmental benefits of cleanups can help with cost-benefit analyses, a key element in identifying productive and attractive revitalization options for stakeholders (Haninger et al., 2017). While this article introduces ecosystem services concepts, it is important to recognize different terminology might be used by different stakeholders when describing benefits that nature and ecosystems provide. Here, we use the term “environmental benefits” as this term is better understood by a wide range of stakeholders.

One primary area of promoting environmental benefits resulting from brownfield cleanups is the application of green design or nature-based elements, ranging from including green spaces to using green infrastructure as part of the remediation work. In general, the incorporation of green spaces and infrastructure elements can take form in a variety of ways from planning open spaces (e.g., landscape design of natural parks) to green roofs, nature trails, and permeable parking lots (Lewis et al., 2018). A green space design element creates an undeveloped piece of land that’s accessible to the community for use as parks, community gardens, and public plazas. These types of open spaces provide recreational areas to enhance the environmental quality and aesthetics of a neighborhood. While green spaces are aesthetically pleasing, they are also an important resource for providing people access to nature while living in cities. For example, a former 200-acre industrial brownfield site in Milwaukee, WI, known as “Milwaukee Road” was redeveloped into an energy efficient, green facility, including providing access to nature. This redevelopment included 70 acres of greenspace for reestablishing pre-industrial ecosystem conditions while also protecting the location from further environmental damage (Lewis, 2008). Within these 70 acres, the community has access to three separate parks, each with different amenities. Chimney Park has two chimney stacks to pay homage to the history of the Milwaukee Road as well as parks space and recreational athletic courts. River Lawn Park provides canoeing and kayaking launches as well as a network of walking and biking trails. The adjacent Airlines Yards Park is 23 acres of natural habitat, native vegetation, and access to 2,600 feet of riverbank (Lewis, 2008). Together these parks provide the community with a variety of opportunities to learn about the city as well as enjoy the environment around them. While green infrastructure approaches encompass a large variety of practices and definitions, the main goal of incorporating green infrastructure elements into this project was to provide economical and effective approaches to support water management efforts for restoring and protecting the natural water cycle. Examples include restoring wetlands, use of bioswales, and planting trees.

The U.S. Environmental Protection Agency (US EPA) estimates that there are over 1 million brownfield sites in the United States (US EPA, 2023b). Accomplishing a successful “Brown-to-Green” project can not only be aesthetically pleasing but also be leading examples in sustainable redevelopment. According to a U.S. Department of Energy survey, the U.S. has over 5.9 million commercial buildings in 2018 (US DOE, 2020), providing opportunities to consider redeveloping spaces into energy efficient “green” building designs, and consideration of other sustainable efforts. Brownfield cleanup initiatives and designs are community specific, often tied to the footprint and history of a given property. As a result, having the community involved in visioning such projects allows them to identify what they want and what would be most beneficial to the community.

Research to address brownfield issues includes development of remediation technologies, risk and environmental assessment, and stakeholder (community and public) engagement and communication (Brebbia, 2006). Recent work has focused on highlighting environmental benefits at brownfield sites, including connections to human health, the community, and surrounding wildlife (Mastervich and Harwell, 2022). The objectives of this article are to introduce concepts of environmental benefits to the cleanup of brownfield sites, explore examples of environmental benefits achieved from brownfield cleanups, and discuss how to bring these types of benefits into brownfield planning efforts.

## 2 Anatomy of brownfield cleanups

### 2.1 Overall process

**FIGURE 1:**
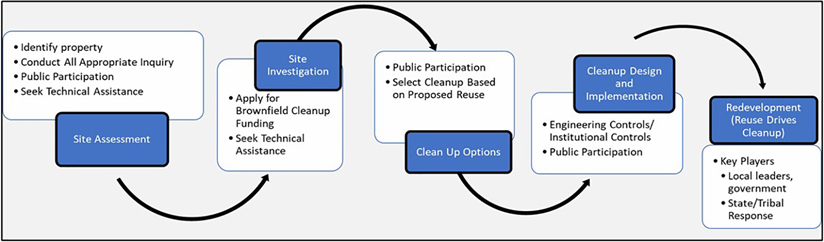
Brownfield cleanup process.

Since 1995, the US EPA’s Brownfield and Land Revitalization Program has focused on a results-oriented program to help communities manage contaminated properties (US EPA, 2023b). The anatomy of a brownfield cleanup is dependent on the site and its contamination. In the conceptual framework of the decision-making process, five steps shown in Figure 1 demonstrate the cleanup process beginning with Site Assessment and ending with elements of Redevelopment. A cleanup begins with an environmental Site Assessment designed to help the community better understand if the current environmental conditions at the property are considered harmful. During this phase, surface soil, water, and sediment samples may be taken to investigate the specific hazards that are present to achieve a better understanding of the type of funding that will be needed for cleanups. A site is not only inspected during a Site Assessment, but investigations are also conducted to help determine who is potentially liable for the contamination on the site. During the Site Investigation phase, the US EPA’s Brownfield program provides direct funding for brownfield assessment, revolving loans, technical assistance, research, training, and cleanup (US EPA, 2023b). Resources from the US EPA Brownfield program, federal partners, and state agencies are identified for the brownfield grant activities, including assessment grants, revolving loan fund grants, cleanup grants, multipurpose grants, job training grants, and the State and Tribal Response Grant program. The Cleanup Options design process uses a risk-based cleanup approach to help determine the level of cleanup needed at a brownfield property. During the Cleanup Design and Implementation phase, the amount of engineering and institutional controls identified for a cleanup depends on the anticipated future plans of reuse for the site. After cleanup, the site is ready for the Redevelopment Phase. Ideally, an economic perspective is also considered when determining the cleanup process to create the most successful redevelopment for the community. Further, initial visioning of future land use can be brought into early aspects of the cleanup process. This can be important for the consideration of sustainable or nature-based remediation solutions into planning and design (e.g., relying on intrinsic capabilities of some plants, bacteria, and fungi to detoxify soils and groundwater) and inform a larger discussion about future green elements and green uses at a given site.

### 2.2 Recent U.S. policy tools encourage enhancing ecosystem services in brownfield revitalization

United States policy initiatives increasingly encourage land revitalization that enhances ecosystem services and applies nature-based solutions. These policy initiatives and related tools provide opportunities to restore and enhance multiple ecosystem services in land revitalization, such as projects in brownfields. The message of the importance of enlisting nature-based solutions to fight climate change is evident in the 2022 White House Office of Science and Technology policy “Opportunities for Accelerating Nature-Based Solutions: A Roadmap for Climate Progress, Thriving Nature, Equity, and Prosperity,” which focuses on enhancing or providing ecological habitats/ecologically-based solutions, including relevant to community development and economic revitalization, that provide multiple ecosystem services while addressing bigger, more comprehensive issues like climate change. This roadmap highlights nature-based solutions as “actions to protect, sustainably manage, or restore natural or modified ecosystems to address societal challenges, simultaneously providing benefits for people and the environment” (White House Council on Environmental Quality, White House Office of Science and Technology Policy, White House Domestic Climate Policy Office, 2022). In 2021 the White House Executive Order, “Protecting Public Health and the Environment and Restoring Science to Tackle the Climate Crisis” (EO 13990), recognizes the value of ecosystem services in accounting for the benefits of reducing climate pollution (White House, 2021). This is relevant to reducing and mitigating toxins in our local ecosystems, which is a core goal of brownfields planning, cleanup, and reuse. Subsequently, in 2022, the Executive Order “Strengthening the Nation’s Forests, Communities, and Local Economies” (EO 14072), references ecosystem services and emphasizes the importance of enlisting nature-based solutions to fight climate change, including the call for reporting nature-based solution opportunities to the National Climate Change Task Force (White House, 2022). Finally, at the time this article was submitted, the US Office of Information and Regulatory Affairs and the Office of Management and Budget released for public comment, “Guidance for Assessing Changes in Environmental and Ecosystem Services in Benefit-Cost Analysis” to assist US Federal agencies specifically in developing their regulatory impact analyses, policy, and program alternatives to include ecosystem services. While this is focused on a US federal nexus, they recognize that the guidance may also be relevant to other rule types “likely to have some ecosystem service effects include rules affecting contaminated site cleanup, financial assistance, trade, fees, royalties, quotas, or credits, among others” (US OIRA and US OMB, 2023). Taken together, these policy tools and initiatives provide conceptual guidance to link brownfields revitalization with goals of enhancing multiple ecosystem services.

### 2.3 Public engagement

One challenge during the redevelopment process involves connecting brownfield redevelopment efforts to the larger community (stakeholders) in working to achieve goals which provide multiple societal benefits, such as public health and environmental protection, creation of jobs, public safety, and city revitalization (McCarthy, 2002). Historically, brownfields efforts focused primarily on providing incentive for developers as opposed to community-centered developments (Pippin, 2008). Community involvement in brownfields projects can promote the goals of revitalization, sustainable development, and smart growth. For example, the New Jersey Brownfields Development Area program recognizes that brownfield remediation should take place with community involvement (New Jersey, 2023). This program, established in 2002, requires state agencies, including the New Jersey Department of Environmental Protection, to work with local communities near brownfield sites to help with design and implementation of remediation plans. There may be local, regional, or state-relevant rules for community involvement in community planning efforts. There are also additional rules for community engagement for community planning responses to physical, social, economic, or governmental impacts by the actions of the Department of Defense (DoD) (24 CFR § 570.401, 1994).

The involvement of a community is crucial to a site’s redevelopment, especially for addressing the community’s needs and desires for the space. Often, the community starts off being involved and interested in a project, but as time progresses public engagement has the potential to diminish unless the project is large enough to have “public actor facilitation, i.e., representatives from public entities devoted time and energy to overcome barriers and complete the redevelopment.” (Yount and Meyer, 1999). Early involvement with the community can help create an understanding of the process to improve buy-in and avoid community objections and litigation. When engaging in the community, using a common language has become an important strategy when talking to citizens from various backgrounds. Without having a common vocabulary, confusion can occur between local community members and the practitioners leading brownfields revitalization. All of these strategies are compatible with, and foundational for, consideration of design elements that add environmental benefits to a redevelopment effort. Specifically, early engagement with the community on the vision of a redevelopment creates the opportunity to identify elements such as green infrastructure that can then be incorporated into plans.

## 3 Elements of ecosystem services relevant to brownfield cleanups

### 3.1 Overview of ecosystem services

Considering the value of ecosystem services in decision-making improves the way resources are managed to benefit the community environmentally, economically, and socially, without negatively impacting essential components of the natural ecological community that may be present. In general, ecosystem services can typically be categorized by four main types: provisioning, regulatory, supporting, and cultural (Millennium Ecosystem Assessment, 2005). Provisioning services maintain the supply of natural products such as fuel, food, timber, water, and soil. An example in a brownfield cleanup context is the creation of a community garden (where appropriate). While provisioning deals with natural products, regulatory services filter pollutants to help water and air quality. Supporting services maintains both provisioning and regulatory services to preserve healthy habitats in both species and genetic diversity. An example in a brownfield cleanup context is the creation of habitat to support pollinator services. Finally, cultural services are intangible benefits of nature, such as spiritual and psychological benefits that include outdoor recreational activities (Holzman, 2012). An example in a brownfield cleanup context is the creation of greenspaces or community parks.

We conducted a non-exhaustive literature survey to identify a suite of nature-based solution design elements that contained resultant ecosystem services benefits. An example of common brownfield redevelopment elements, grouped into major categories of community garden, green infrastructure, greenspace, native plants, outdoor classroom opportunities, and educational signage, resulting in examples of environmental benefits is shown in Table 1 (Seybr and Lewis, 2008; Rall et al., 2015; Chowdhury et al., 2020). Community gardens can provide a source of food but cannot be established until soil testing demonstrates successful contaminant removal. Green infrastructure is used to help with stormwater management; however, local site conditions may favor routing water around a site, rather than through it. Other stormwater management features may include bioswales, rainwater harvesting, permeable pavements, or green roofs. Green roofs and green walls can provide heat attenuation and air cleaning benefits. Rain gardens and native plants can provide wildlife habitat as well as pollinator benefits. Education benefits can be provided through environmental signage and using spaces for classroom opportunities.

A number of greenspace features (e.g., community parks, green corridors, playgrounds, walking trails, and waterfront access) can provide myriad recreational benefits. With ongoing global growth and complexity of urban areas, the pressure to develop open green spaces within cities is increasing (Washbourne et al., 2020). By implementing ecological restoration of these contaminated sites (also referred to as eco-revitalization; US EPA, 2009), this recovery returns lost ecological function to previous environmental benefits. The use of greenspace and green infrastructure on brownfield land is an important mechanism for restoring sites, especially in the surrounding urban environment. Developing greenspace and green infrastructure can increase property value in and improve the quality of life of the surrounding community (Amati and Taylor, 2010).

### 3.2 Examples

Environmental benefits can be realized across a range of contaminants depending on the conditions of the site (Table 2). Depending on the end goal of the remediation process, the amount of cleanup required varies.

Examples of brownfield cleanup elements with environmental benefits can be seen in a variety of ways (Table 3). The authors identified three examples to described in more detail. Each example was reviewed by at least two authors to identify the environmental benefits elements described below.

**FIGURE 2:**
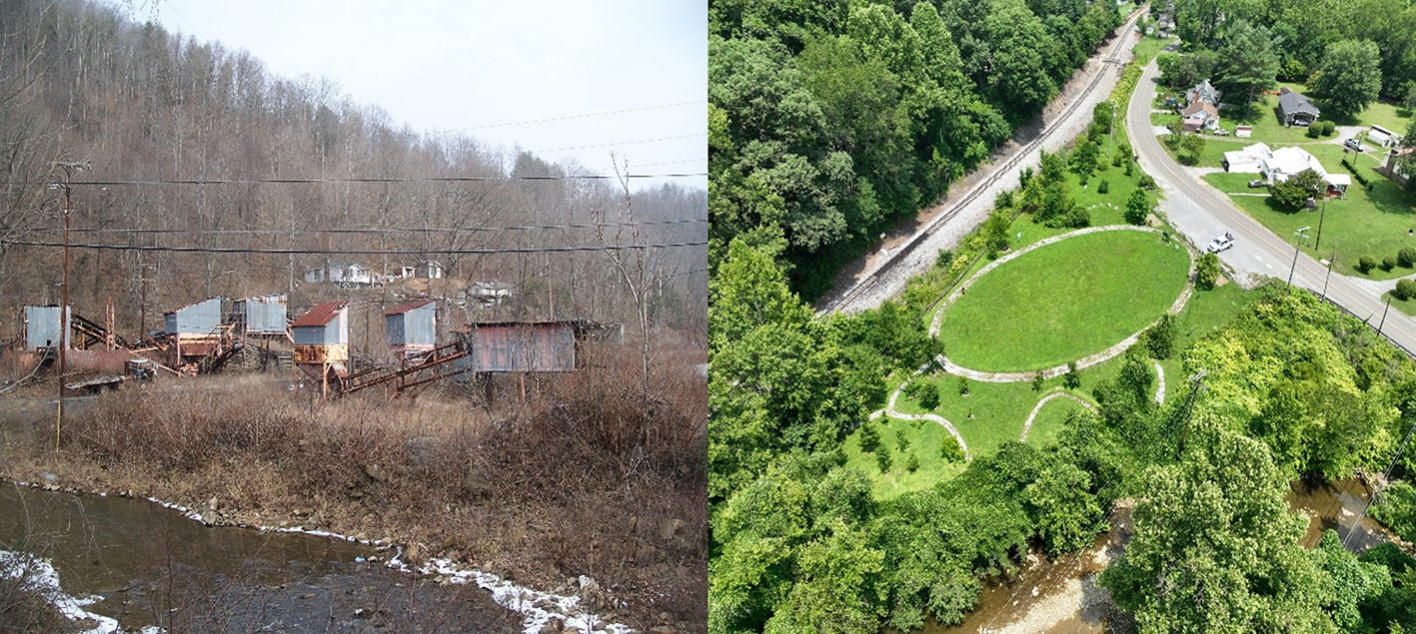
Stone Creek Tipple Site before (left) and after (right) cleanup. Photo credits: Virginia Department of Environmental Protection.

The Stone Creek Tipple Site in Pennington, VA was once a mine-scarred land that was causing environmental and economic problems. This site was cleaned up and revitalized by removing the coal deposits; once the deposits were removed, two feet of soil was added. The transformation continued by seeding the land with native plants and stream bioengineering. The Pennington community can now enjoy trails, learning stations, and recycled material benches throughout this once contaminated land (Figure 2).

At a former abandoned Hi-Tech gas station in Brandywine Village (Wilmington, DE) the community was involved with the redevelopment process (as seen in Table 3). A permeable parking lot was constructed from previous paving material which enabled water to collect in piping under the parking lot from where it is transported to a bioswale. The project put peat moss and sand below the bioswale and parking lot to help absorb any contaminants that may be transported from the runoff.

**FIGURE 3:**
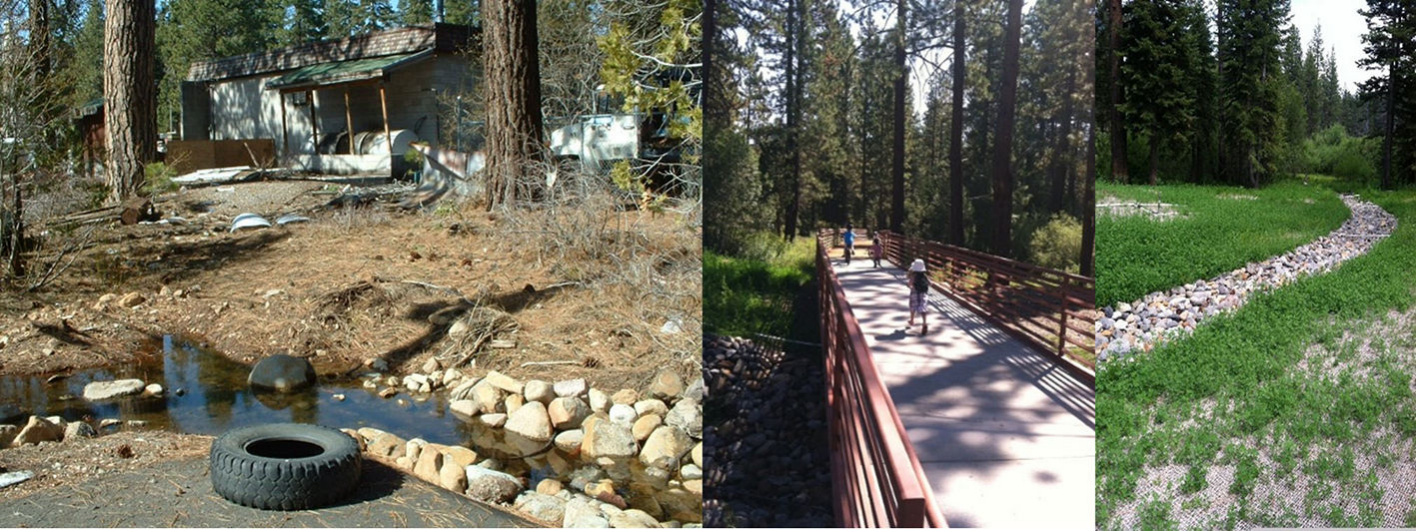
Snow Creek Wetlands restoration project before (left) and after (middle, right) cleanup. Photo credits: Kansas McGahan (left) and Placer County (middle, right).

The Snow Creek Wetlands restoration project in Tahoe Vista, CA, was a brownfields site that required cleanup and focused on features that provided ecosystem services, community benefits, and citizen engagement (Schuster et al., 2014). The 3.5-acre site is a filled-in wetland that used to be a former concrete plant site (Figure 3). With its location adjacent to a tributary of Lake Tahoe and close to the edge of the lake, the project team wanted to do more than just remediate the concrete material, address high pH in the soil, and remove the hydrocarbon-contaminated fill. The multi-stakeholder team was interested in environmental benefits of water quality improvements, access to greenspace, and improvement in aesthetics. The stakeholder-driven design process identified additional environmental benefits, including following the Placer County Low Impact Development Guidebook (Placer County, 2012) in designing restoration of the stream zone and upland habitat, establishing a multi-use path that provided access to nature, and installation of environmental education signage (Figure 3). Land revitalization in this project aimed to restore hydrology and create a buffer for existing environmentally sensitive areas and nearby residential neighborhoods. The team used the infrastructure-oriented Envision^™^ Sustainability Rating Tool (Institute for Sustainable Infrastructure, 2023) and emphasized sustainability practices, including working to reuse and repurpose existing site materials, consideration of climate change resiliency in the design process, the use of extensive stakeholder engagement, community involvement through educational outreach, and consensus-driven decision-making.

## 4 Tools and resources to enhance multiple benefits in brownfield cleanups

Multiple decision support tools and frameworks have been created for analysis of environmental benefits for different decision-making contexts. In a brownfield cleanup context, decision support tools can be used to overcome challenges among different stakeholder values and goals and to maintain interest in the revitalization effort over time. We conducted a non-exhaustive survey for “ecosystem services” and “environmental benefits” tools potentially useful for one or more steps in a generic brownfields cleanup process. For brownfields, example decision support tools that may be useful to a community include the Vision-to-Action tool, Timbre Brownfield Prioritization Tool, the Envision Sustainability Rating Tool, EnviroAtlas, the Eco-Health Relationship Browser, and the Final Ecosystem Goods and Services (FEGS) Scoping Tool. Each have been developed to address the different aspects of environmental decision-making processes. We note that there may be other applicable tools for consideration.

The Vision-to-Action tool^18^ is used by the US EPA to aid the stakeholder community. This tool is designed to inspire community members to visualize what they value, how they would like to see their community change, and to bring those visualizations into reality (US EPA, 2022). The tool’s process brings together ideas from multiple individuals/stakeholders graphically to try to form common themes that lend themselves to dedicated decisions/actions by community leaders. The overview of the Vision-to-Action tool includes several examples of community and waterfront revitalization (US EPA, 2022).

The Timbre Brownfield Prioritization Tool^19^ is used by stakeholders to identify which brownfield sites should be considered for redevelopment. The tool prioritizes the success factors determined by the stakeholder engagement and a site’s redevelopment potential. The Timbre Brownfield Prioritization Tool was tested on 235 European brownfield sites across the Czech Republic, Germany, and Poland (Pizzol et al., 2016). The authors concluded that the tool was effective in providing a starting point for users who need to collect detailed information for preselecting those sites that had the highest potential for redevelopment. They also concluded that during pre-selection of brownfield sites the tool was useful in identifying agricultural brownfields with the highest redevelopment potential based on pre-determined criteria. Overall, the Timbre Brownfield Prioritization Tool provides a better understanding of the different variables when choosing a site based off the relevant parameters in line with the user’s needs.

The Envision Sustainability Rating Tool^20^ is a tool for identifying sustainable elements used during planning, design, and construction of infrastructure projects (Institute for Sustainable Infrastructure, 2023). Elements are grouped into five categories (quality of life, leadership, resource allocation, natural world, and climate and risk). The rating system, developed by The Institute for Sustainable Infrastructure (ISI), includes a self-assessment, verification, and award recognition components. The Snow Creek Wetlands restoration project described above used the Envision Sustainability Rating Tool.

The EnviroAtlas^21^ is an interactive, web-based map resource, providing over over 400 map layers layers incorporating seven broad datasets to organize information on potential environmental benefits. This tool can be used to help screen and evaluate a community’s vulnerabilities as well as assets which can give an indication on how to redevelop a brownfield site (US EPA, 2023a). Recently, the EnviroAtlas released a set of brownfield-relevant, curated map layers, allowing users to view and assess brownfield information for any location in the conterminous United States.

The Eco-Health Relationship Browser^22^ is an interactive tool that provides information connecting ecosystems and the benefits they provide to how those benefits affect human health and the community (Jackson et al., 2013). This tool has six broad urban ecosystem categories including: water hazard mitigation, recreation and physical activity, heat hazard mitigation, water quality, air quality, and aesthetics and engagement with nature. Each of these categories can be explored to learn about research demonstrating direct human health connections.

The Final Ecosystem Goods and Services Scoping Tool^23^ is a tool for decision makers early in the project stage. The FEGS Scoping Tool prioritizes stakeholders, environmental attributes, and beneficiaries in the restoration context. By identifying the more relevant ecosystem services, the decision-making process will make restoration in the community more effective. The intended audience for this approach includes communities, private and public sectors, non-profits, and individuals.

An evaluation of environmental benefits can include examining loss of benefits resulting from the impacts of the brownfield site, neighboring environmental benefits that could be enhanced by cleanup, or identification of potential future environmental benefits as part of brownfield cleanup and reuse design. An organizational way to explore these resources is to crosswalk the generic brownfields cleanup phases (Figure 1) with potential tools and resources as outlined in Table 4. For early cleanup phases of environmental site assessment and site investigation, a primary consideration is the evaluation of a community’s vulnerabilities and assets along with relevant environmental elements that may be involved in a given brownfield cleanup decision. Consideration of environmental benefits can also be incorporated into a community’s future visioning of cleanup options.

## 5 Discussion and summary

Nature-based solutions are actions to protect, sustainably manage, or restore natural or modified ecosystems to address societal challenges, simultaneously providing benefits for people and the environment. Concepts from the field of ecosystem services, benefits from nature, can be useful for communities and decision-makers to establish priorities for brownfield development projects which identify and prioritize linkages between the environment and human welfare. This article introduced concepts of enhancing environmental benefits in brownfield sites, explored examples of environmental benefits achieved from brownfield cleanups, and discussed how to consider these types of benefits into brownfield planning efforts.

Decision-makers can use recent policy tools to encourage brownfields revitalization that helps to combat the climate crisis, apply nature-based solutions, and enhance ecosystem services. Operationalizing the concepts and tools presented in this study will increase the number of resources available to communities to help them design and implement their brownfield reuse goal that may include environmental benefits. As more brownfield applications use the tools described here, these decision support tools can be better refined and translated for use in brownfield applications in future efforts.

While Section 2.1 describes the generic cleanup process for United States brownfield sites, our study is also relevant to cleanups elsewhere. As of 2018, there were approximately 2.8 million potentially contaminated sites in the European Union (Pérez and Eugenio, 2018), for which recent efforts have focused on mechanisms such as the pending European Soil Monitoring Law (COM, 2021) to establish connections between site cleanups and ecosystem services. The concepts described here can be used to enhance other efforts such as the European Union’s GREEN SURGE effort, looking to connect urban green spaces, green infrastructure, and ecosystem services (Rall et al., 2015) and environmental elements of low impact design for brownfields cleanup in New Zealand (Seybr and Lewis, 2008).

Future activities should focus on communication and operationalizing these concepts, the application of tools and other resources in on-the-ground applications, and documenting and communicating successful examples. Developing a library of examples has been successfully used in green and sustainable remediation efforts and can result in extending these ecosystem services concepts to a wider audience, including natural and social scientists, communities, and environmental decision-makers. Finally, over time, a larger suite of “success stories” can be documented and shared by including consideration of environmental benefits in brownfield cleanups.

## Figures and Tables

**TABLE 1:** Examples of common nature-based solutions, often called design elements, and the associated ecosystem services and general timeline to achieve benefits (taken from Seybr and Lewis, 2008; Rall et al., 2015; Chowdhury et al., 2020).

Nature-Based Solutions (Design Elements)	Example Ecosystem Services	Timeline (Years)
**Community Garden**	Food production	1–3
**Green Infrastructure**	Stormwater & flood control	1+
**Bioswales**	Stormwater & flood control	1
**Green Roofs**	Stormwater & flood control Temperature regulation	2–5
**Permeable Pavements**	Flood protection	Immediate
**Rain Gardens & Rainwater Harvesting**	Wildlife habitat Stormwater & flood control	Immediate
**Greenspace^1^**	People accessing nature	1+
**Community Park**	Recreation benefits	Immediate
**Green Corridors**	Noise buffering Wildlife habitat	1+
**Playground**	Recreation benefits	Immediate
**Walking Trails**	Health and Recreation benefits	Immediate
**Waterfront Access**	Recreation benefit	Immediate
**Native Plants**	Pollination benefits	1+
**Outdoor Classroom**	Environmental education	Immediate
**Educational Signage**	Environmental education	Immediate

For examples of brownfields revitalization that successfully incorporated ecosystem services elements, see Table 3.

**TABLE 2:** Examples of land use changes after brownfield cleanup of different types of contaminants and hazardous materials.

Example of Land Use Changes After Cleanup	Types of Contaminants & Hazardous Materials Remediated
The rails-to-trails project in Brea (CA) created 4.5 miles of bike and pedestrian trails^2^	Arsenic
Cleanup and rehabilitation of the Ball Park in McGill (NV)^2^	Asbestos
An abandoned urban lot is now the Emerson Street Garden in Portland (OR)^3^	Lead
5-acre cleanup along Spicket River (Lawrence, MA) turned into suite of environmental benefits^4^	Mercury
Redevelopment of former gas stations to create greenspace gateways into towns across Colorado^5^	Petroleum and Hydrocarbons
Riverbend Park remediation resulted in recreational benefits (Medford, MA)^6^	Polycyclic Aromatic Hydrocarbons (PAHs)
Turned a burn dump into a 9-acre Cooley Landing Park (East Palo Alto, CA) with native plants and grasses^2^	Polychlorinated Biphenyls (PCBs)
After understanding former property use made decision to turn into a garden (Manhattan, KS)^7^	Pesticides/Herbicides

Each case study reference (see footnotes) was reviewed by at least two authors to identify the environmental benefits elements listed in the left column.

**TABLE 3:** Examples of brownfield cleanup efforts that included design elements to create or enhance ecosystem services.

Location	Previous Land Use	Current Land Use	Ecosystem Services
Pennington Gap, VA	Stone Creek Tipple Site Coal mine^8^	Community park & outdoor classroom	Enhancing wildlife habitat; Stormwater and flood control; Recreational benefits; Pollinator benefits; Environmental education; Access to nature
Wilmington, DE	Brandywine Village Abandoned Hi-Tech gas station^9^	Greenspace (permeable parking lot)	Stormwater & flood control
Tahoe Vista, CA	Snow Creek Stream Environment Zone (SEZ) project^10^	Restored wetlands	Recreational benefits; Environmental education; Access to nature; Climate change resiliency
Palmerton, PA	Palmerton Zinc Pile^11^	450-acres of Native Prairie & Lehigh Gap Nature Center	Pollinator benefits; Environmental Education; Recreational benefits
Town of Coventry, RI	Former dumping ground^12^	20-acre Sandy Acres Recreation Area	Enhancing wildlife habitat; Stormwater and flood control; Recreational benefits
Crab Orchard, KY	Lincoln Co. Scrap Metal^13^	Public park	Walking trails; Recreation benefits
Tillamook County, OR	Veneer Mill^14^	Protected wetland	Wildlife habitat; Stormwater and flood control
Lake Tahoe, CA	Concrete Plant^15^	3-acre parcel of land with native habitat	Enhancing wildlife habitat; Access to nature
Brea, CA	Abandoned Union Pacific Railroad^16^	Multi-use trail	Access to nature; Environmental education; Recreational benefits
Fitchburg, MA	Rubber Factory^17^	Riverfront Park	Enhancing wildlife habitat; Stormwater & flood control; Recreational benefits

Each case study reference (see footnotes) was reviewed by at least two authors to identify the ecosystem services elements listed in the right column. For a list of common environmental benefits/ecosystem services in brownfield cleanup design, see Table 1.

**TABLE 4:** Cross-walking different brownfield cleanup phases with tools and other considerations for a community to identify elements with environmental benefits.

Cleanup Phase	Description	Tools & Considerations
Environmental Site Assessment	Investigate specific hazards that are present to achieve a better understanding of how to approach the cleanup	When developing cleanup and reuse goals, evaluate a community’s vulnerabilities and assets, including environmental elements. Example resources: The Vision-to-Action tool; Timbre Brownfield Prioritization Tool; Envision Sustainability Rating Tool; EnviroAtlas; and EcoHealth Relationship Browser
Site Investigation	Identify funding sources	
Cleanup Options	Determine level of cleanup and type of project elements	Consider environmental benefits when a community develops a vision for a site’s reuse to inform options for project elements and options. Example resources: EnviroAtlas; FEGS Scoping Tool; Envision Sustainability Rating Tool
Cleanup Design and Implementation	Type of cleanup and anticipated future reuse plans defines engineering and institutional controls	
Redevelopment Phase	Integrates site assessments and incorporate neighborhood-relevant features to address the community concerns for the reuse vision	Example resources: The Vision-to-Action tool; Envision Sustainability Rating Tool

## Data Availability

The original contributions presented in the study are included in the article. Further inquiries can be directed to the corresponding author.

## Footnotes

1 Note other design elements in this table are also greenspaces

2 https://www.epa.gov/sites/default/files/2015-10/documents/epa_oblr_successstory_region9_openspace_v2_508.pdf. Accessed January 21, 2024.

3 https://www.epa.gov/sites/default/files/2015-09/documents/bf-ss-emerson-street-032911.pdf. Accessed January 21, 2024.

4 https://www.epa.gov/sites/default/files/2015-09/documents/bf_ss_gw_lawrence_urban_waters_033111_web.pdf. Accessed January 21, 2024.

5 https://neiwpcc.org/wp-content/uploads/2020/06/FINAL_LUSTLine-87_Web.pdf. Accessed January 21, 2024.

6 https://medfordenergy.org/gogreen/brownfields/. Accessed January 21, 2024.

7 https://www.gardeningonbrownfields.org/docs/MF3096 Historical Property Usage and Implications 2017 update.pdf. Accessed January 21, 2024.

8 https://www.njit.edu/tab/sites/njit.edu.tab/files/stone_creek_outdoor_classroom_and_community_park.pdf. Accessed January 21, 2024.

9 https://www.njit.edu/sites/njit.edu.tab/files/de-wilmington.pdf. Accessed January 21, 2024.

10 https://www.placer.ca.gov/1657/Snow-Creek-Stream-Environment-Zone-Resto. Accessed January 21, 2024.

11 https://cumulis.epa.gov/supercpad/SiteProfiles/index.cfm?fuseaction=second.Cleanup&id=0300624#bkground. Accessed January 21, 2024.

12 https://www.epa.gov/sites/default/files/2016-01/documents/epa_oblr_successstory_sandyacres_v5_508.pdf. Accessed January 21, 2024.

13 https://19january2017snapshot.epa.gov/land-revitalization/lincoln-co-scrap-metal-crab-orchard-kentucky.html. Accessed January 21, 2024.

14 https://www.epa.gov/sites/default/files/2019-12/documents/going_with_the_flow_to_prevent_flooding.pdf. Accessed January 21, 2024.

15 https://www.epa.gov/sites/default/files/2015-10/documents/epa_oblr_successstory_region9_openspace_v2_508.pdf. Accessed January 21, 2024.

16 https://www.ci.brea.ca.us/791/The-Tracks-at-Brea-Trail. Accessed January 21, 2024.

17 https://archive.epa.gov/region1/brownfields/web/html/fitchburg_hrf_agp.html. Accessed January 21, 2024.

18 https://www.epa.gov/brownfields/brownfields-vision-action-tool. Accessed January 21, 2024.

19 http://www.timbre-project.eu/tl_files/timbre/Intern/4%20Work%20Packages/WP3/Presentations&Manuals/Brochure_TIMBRE_Prioritisation_Tool.pdf. Accessed January 21, 2024.

20 https://sustainableinfrastructure.org/envision/use-envision/. Accessed January 21, 2024.

21 https://www.epa.gov/enviroatlas/enviroatlas-brownfields. Accessed January 21, 2024.

22 https://www.epa.gov/enviroatlas/enviroatlas-eco-health-relationship-browser. Accessed January 21, 2024

23 https://www.epa.gov/eco-research/final-ecosystem-goods-and-services-fegs-scoping-tool. Accessed January 21, 2024.
